# The Effects of Chain-Extending Cross-Linkers on the Mechanical and Thermal Properties of Poly(butylene adipate terephthalate)/Poly(lactic acid) Blown Films

**DOI:** 10.3390/polym13183092

**Published:** 2021-09-14

**Authors:** Juliana V. C. Azevedo, Esther Ramakers-van Dorp, Berenika Hausnerova, Bernhard Möginger

**Affiliations:** 1 Faculty of Technology, Tomas Bata University in Zlin, Vavreckova 275, 760 01 Zlin, Czech Republic; Juliana.Azevedo@bio-fed.com; 2 Department of Natural Sciences, University of Applied Sciences Bonn-Rhein-Sieg, von Liebig Str. 20, 53359 Rheinbach, Germany; Esther.vanDorp@h-brs.de (E.R.-v.D.); Bernhard.Moeginger@h-brs.de (B.M.); 3 BIO-FED, Branch of AKRO-PLASTIC GmbH, BioCampus Cologne, Nattermannallee 1, 50829 Köln, Germany; 4 Centre of Polymer Systems, University Institute, Tomas Bata University in Zlin, Nam. T.G. Masaryka 5555, 760 01 Zlin, Czech Republic

**Keywords:** chain extenders cross-linker, polybutylene adipate terephthalate, polylactic acid, blow molding, thermo-mechanical properties

## Abstract

This study investigates the effects of four multifunctional chain-extending cross-linkers (CECL) on the processability, mechanical performance, and structure of polybutylene adipate terephthalate (PBAT) and polylactic acid (PLA) blends produced using film blowing technology. The newly developed reference compound (M·VERA^®^ B5029) and the CECL modified blends are characterized with respect to the initial properties and the corresponding properties after aging at 50 °C for 1 and 2 months. The tensile strength, seal strength, and melt volume rate (MVR) are markedly changed after thermal aging, whereas the storage modulus, elongation at the break, and tear resistance remain constant. The degradation of the polymer chains and crosslinking with increased and decreased MVR, respectively, is examined thoroughly with differential scanning calorimetry (DSC), with the results indicating that the CECL-modified blends do not generally endure thermo-oxidation over time. Further, DSC measurements of 25 µm and 100 µm films reveal that film blowing pronouncedly changes the structures of the compounds. These findings are also confirmed by dynamic mechanical analysis, with the conclusion that tris(2,4-di-tert-butylphenyl)phosphite barely affects the glass transition temperature, while with the other changes in CECL are seen. Cross-linking is found for aromatic polycarbodiimide and poly(4,4-dicyclohexylmethanecarbodiimide) CECL after melting of granules and films, although overall the most synergetic effect of the CECL is shown by 1,3-phenylenebisoxazoline.

## 1. Introduction

The global plastic waste problems have positively affected the development of biopolymers and other sustainable materials [1,2,3,4] towards the substitution of traditional packaging plastics such as polyethylene and polystyrene with biodegradable starch and biopolyesters [5,6,7]; however, for more demanding engineering applications, their mechanical properties must be enhanced without compromising their biodegradability. To accelerate this conversion, the properties of the biomaterials must be enhanced without compromising their biodegradability. Many biopolymers are combined with other polymers to balance the properties required for specific applications. Compounding is used to make a biopolymer fit for specific applications by blending the biopolymer with other polyesters and adding mineral fillers to provide optimized properties at satisfactory prices.

Polybutylene adipate terephthalate (PBAT) [8,9,10,11] and polylactic acid (PLA) [12,13,14,15,16,17] have the highest market shares. PLA is both biodegradable and entirely renewable as it originates from starch. It is film blowable, making it interesting for consumer packaging, although it suffers due to its limitations in terms of its brittleness during film blowing and its hygroscopic behavior [18,19,20,21,22,23,24,25]. PBAT is a random copolymer of butylene adipate and terephthalate, which has shown to be fully biodegradable when composted [26,27]. This polymer owes its biodegradability to the butylene adipate groups and its stability and mechanical properties to the terephthalate groups. Due to the random structure of the copolymer, PBAT generally cannot crystallize to a significant degree; hence, it has a wide melting peak, unlike that of either the aliphatic or aromatic homopolymer–polybutylene adipate or polybutylene terephthalate, respectively [8,9,10,11]. Due to its low crystallinity, PBAT has low storage moduli [18,19,20,21,22,23,24,25]. In order to increase the mechanical and barrier behaviors, it is often compounded with inorganic fillers such as calcium carbonate and talc [28,29].

Recent developments in the field of chain-extending cross-linkers (CECL) offer chances to improve the melt strength, thermal stability, and phase compatibility of polymer blends; however, there are only a few investigations of CECL in PBAT/PLA25 [30,31,32,33]. Al-Itry et al. [25] investigated the thermostability of PLA and PBAT after processing and the resulting effects on the structural, rheological, mechanical, and morphological properties of PLA, PBAT, and their blends compatibilized with Joncryl© ADR 4368 (BASF). The results showed that PLA and PBAT degraded under temperature due to decreased molecular weight and intrinsic viscosity. The incorporation of Joncryl© improved the thermal stability due to the epoxy reactive functions and increased the molecular weight, intrinsic viscosity, shear thinning, and elasticity during the processing. Wang et al. [30] found that an epoxy-terminated branched polymer (ETBP) enhanced the interfacial compatibility and mechanical properties of PLA/PBAT. An ETBP content of 3.0 phr increased the elongation at the break of PLA/PBAT blends from 45 to 270% and the impact strength from 26.2 to 45.3 kJ m^−2^. DMA measurements showed that the difference between the *T_g_* values of PLA and PBAT decreased with increasing ETBP content, indicating that ETBP promotes the interfacial compatibility of PLA and PBAT phases. Dong et al. [31] investigated the phase morphology, mechanical properties, thermal properties, and hydrolytic degradation of PLA and PLA/PBAT blends with and without chain extenders. Chain extenders containing multiepoxy groups improved both the phase compatibility and mechanical properties. The chain-extending effect was confirmed by an increase in molecular weight. Elongations of the break of PLA/PBAT blends were increased from 200% to 500% without compromising the tensile strengths. Arruda et al. [32] investigated 40:60 and 60:40 PLA/PBAT blends with Joncryl© also added as a chain extender and found that both PLA and PBAT showed improved thermal stability and increased complex viscosity; however, they also found a preferential reactivity with the PLA component. Pan et al. [33] studied melt-compounded PLA/PBAT blends using methylene diphenyl diisocyanate (MDI) as a chain extender. Increasing the MDI content led to a higher yield, tensile strength, storage modulus, and elongation at the break associated with yielding deformation between the PLA and PBAT phases. Supplemented DSC, WAXS, and SAXS studies suggested that amorphous PLA and PBAT can be well compatibilized with MDI.

Currently, little is known about how multifunctional CECL affect the compounding of PBAT/PLA blends; therefore, the objective of this study is to investigate the effects of various CECLs on the mechanical, thermal, and long-term aging properties of PBAT/PLA blends in order to optimize their processability and usage performance.

## 2. Materials and Methods

The four CECL with fractions of 1 wt.% were compounded into the reference PBAT/PLA (REF) compound M·VERA^®^ B5029 [34] from BIO-FED, a branch of AKRO-PLASTIC GmbH, Germany, which is used for packaging (fruit and vegetable bags, labels) and agricultural applications:

V1-tris(2,4-di-tert-butylphenyl)phosphite, Songnox^TM^ 1680 (Songwon Industrial Co, Ulsan, South Korea) [35];

V2-1,3-phenylenebisoxazoline, 1,3-PBO powder (Evonik, Essen, Germany) [36];

V3-aromatic polycarbodiimide, Stabaxol^®^ P110 (Lanxess, Cologne, Germany) [37];

V4-poly(4,4-dicyclohexylmethanecarbodiimide), Carbodilite^TM^ HMV-15CA (Nisshinbo, Tokyo, Japan) [38].

The polymer ratio of PBAT/PLA in M·VERA^®^ B5029 is 76% PBAT and 24% PLA. This compound contains a standard formulation and serves well as a reference for the investigation of CECL in packaging applications. The CECLs were chosen after a market study on the currently available additives, which have not been investigated in PBAT/PLA blends.

All ingredients were uniformly mixed using a Mixaco CM 150-D (Mixaco Maschinenbau, Neuenrade, Germany) and compounded in a twin-screw extruder (FEL 26 MTS, Feddem GmbH, Sinzig, Germany) with 26 L/D, a screw speed of 260 rpm, and an output rate of 20 kg h^−1^. The films were blow-molded using an LF-400 (Labtech Engineering Company, Samutprakarn, Thailand) machine with an extrusion temperature of 165 °C and blow-up-ratio of 1:2.5 for both 25 µm and 100 µm films. From an extrusion gap of 0.8 mm, the draw ratios were estimated to be 12 to 14 (25 µm films) and 3 to 4 (100 µm films). The extrusion pressures were 240 bar (REF blend), 290 bar (V1), 159 bar (V2), 230 bar (V4), and 313 bar (V4). Storage before testing occurred for 24 h at 23 °C/50% r.h.

### 2.1. Tensile Properties

The Young’s modulus, tensile strength, and fracture strain were determined according to ISO 527-3 using a tensile testing machine (2.5 kN Zwicki, Zwick Roell, Ulm, Germany) at 23 °C/50% r.h. and a cross-head speed of 200 mm min^−1^. The specimens measuring 170 mm in length and 15 mm in width were examined in the extrusion direction (ED) and transverse direction (TD).

### 2.2. Tear Properties

An Elmendorf tear tester [39] was used to determine the rupture force *F*_break_ (N) and tear resistance *F*_tear_ (N.mm^−1^) at 23 °C/50% r.h. according to ISO 6383-2 for at least 10 specimens (*n* = 10) with measuring 63 ± 0.5 mm in length and 76 ± 0.5 mm in width. Samples were cut out of 25 µm films in ED and TD directions with an incision measuring 20 ± 0.5 mm.

### 2.3. Seal Strength

**Figure 1:**
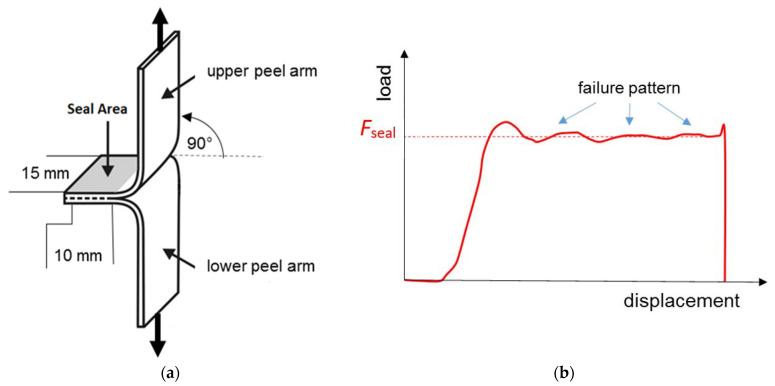
*T*-test sample for seal strength measurements (**a**). DIN 55529 and scheme of a load displacement curve with determination of a seal strength (**b**).

The seal strength tests were performed according to DIN 55529 using a tensile testing machine (2.5 kN Zwicki) with a clamp distance of 40 ± 5 mm and a trigger angle of 90° (Figure 1a). The seal strength is given by the plateau force *F_seal_* (Figure 1b). Samples (200 mm × 200 mm) of 25 µm films were sealed at 90° with respect to the ED using a sealer (SGPE 3000, Willi Kopp e.K., Reichenbach an der Fils, Germany) at 140 °C and 130 N for 3 s with a seal width of 10 mm. Then, stripes with a width of 15 mm were cut out and stored for 24 h at 23 °C/50% r.h.

As cross-linking or chain reactions occur at elevated temperatures, aging tests were performed at 50 °C for 1 and 2 months with subsequent mechanical testing after conditioning for 24 h at 23 °C/50% r.h.

### 2.4. Dynamic Mechanical Analysis (DMA)

The storage modulus *E*’ and loss modulus *E”* were measured in tension mode (displacement controlled) using a DMA 242 E Artemis instrument (Netzsch, Selb, Germany). Samples (7 × 5.9) mm^2^ were punched out of 100-µm-thick films in ED and TD directions. The 25 µm films could not be measured due to low forces. Measurements were performed at a frequency of 1 Hz, heating rate of 2 K min^−1^, deformation amplitude of 20 μm, and preload of 0.2 N over the temperature range of 70 °C to 80 °C.

### 2.5. Melt Volume Rate (MVR)

MVR values were measured using a melt flow indexer (MI-3, Göttfert, Buchen, Germany) according to ISO 1133 at 190 °C with a weight of 2.16 kg. Prior to the measurements, the granules were dried in an IR oven (HB43-S, Mettler Toledo, Greifensee, Switzerland) for 20 min at 110 °C.

### 2.6. Differential Scanning Calorimetry (DSC)

DSC experiments were performed using a DSC Diamond instrument (Mettler Toledo, Switzerland) for granules and a DSC 214 Polyma instrument (Netzsch Gerätebau GmbH, Selb, Germany) for films in standard Al pans in three steps, namely a first heating step, a cooling step, and a second heating step. The measuring conditions were:

Sample weight *m_s_*: (6 ± 1) mg;

Starting temperature *T*_start_: 0 °C;

End temperature *T*_end_: 200 °C;

Heating/cooling rate: 10 K min^−1^;

Repetition: 2;

Atmosphere: N_2_, 20.0 mL 10 min^−1^.

The thermal quantities such as the glass temperatures of hard segments *T_g,hs_*, melting temperature of PBAT *T_m_*_1_, melting temperature of PLA *T_m_*_2_, melting enthalpy of PBAT Δ*H_m_*_1_, melting enthalpy of PLA Δ*H_m_*_2_, crystallization temperature *T_cr_*, and crystallization enthalpy Δ*H_cr_* were evaluated from DSC curves according to ISO 11357-3:2018.

## 3. Results and Discussion

Both the addition of CECL and aging for 1 and 2 months at 50 °C caused changes in the Young’s modulus *E*, tensile strength *σ_max_*, elongation of the break *ε_break_*, tear resistance *F_tear_* in the ED and TD, seal strength *F_seal_* in the ED, and the MVR, indicating the flowability of the blends (Table 1 and Table 2).

Due to the film blowing process, the mechanical properties were anisotropic. The initial modulus and initial tear resistance in the ED were roughly twice those in the TD for the unmodified blend (REF), whereas the elongations of the break coincided with respect to the standard deviations (STD) (Table 1). Thermal aging reduced the tensile strengths by 10 and 30% after 1 and 2 months, respectively. The seal strength dropped by 30%, whereas the modulus, elongation of the break, and tear resistance remained constant with respect to the STD (Table 2). The increases in MVR by 20 and 40% after 1 and 2 months, respectively, indicated chain scission or degradation of polymer chains, resulting in decreased tensile strength and seal strength.

The introduction of CECL modified the properties of the REF. The MVR data showed that V1 and V2 led to chain scission or degradation (MVR increased by more than 25%), whereas V3 and V4 led to cross-linking (MVR decreased by at least 75%). A reinforcing effect (more than 25% increase in stiffness and strength) was observed for V4. The elongations of the break in the ED decreased by at least 40%. This was in accordance with a similar decrease in the tear resistance in the TD, as the stress at the incision tip in the TD acts in the ED. Elongations of the break in the TD and tear resistance in the ED were not altered with respect to the REF. The seal strengths increased for all CECL except for V2 and V4 by more than 25%.

Aging for 1 and 2 months at 50 °C caused characteristic property changes in the REF (Table 2). The MVR showed continuing chain scission or degradation for V1 and V2, whereas V3 and V4 continued to cross-link, further increasing the strength in the ED and TD after 1 and 2 months. Interestingly, V2 also exhibited increased strength in the ED and TD after 1 month. A stronger modulus increase was observed for V4 in the ED after 2 months. The elongations of the break in the ED decreased due to aging being pronounced for V3 and V4 after 1 month and for all compounds after 2 months. This was in line with the decreasing tear resistances in the TD, especially after 2 months. The elongations of the break in the TD were not greatly affected by aging and varied in an arbitrary manner. Interestingly, the tear resistances in the ED increased for all compounds in a pronounced manner, except for V4 after 1 month. The seal strengths decreased for V1, V3, and V4 to close to “zero”, indicating a complete loss of the sealing capability after 2 months. Only V2 showed increases of the seal strength in the range of 10 to 25%.

**Figure 2:**
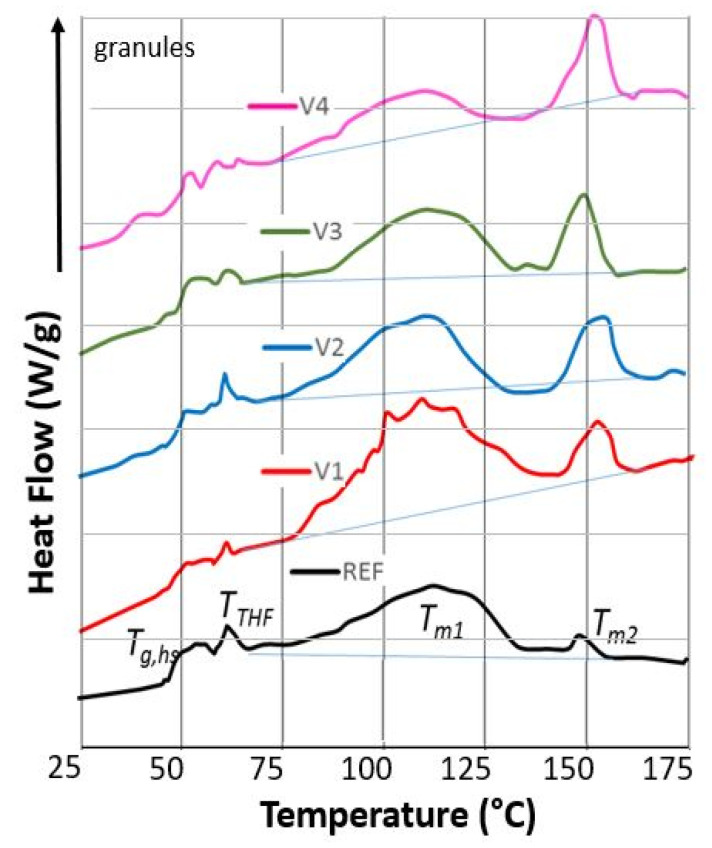
DSC traces of REF, V1 to V4, first runs (0.2 W/g) of granules with glass temperature *T_g,hs_*, the THF boiling temperature *T_THF_*, melting temperatures *T_m_*_1_ and *T_m_*_2_, and corresponding melting and crystallization peaks.

CECL-induced structural changes were investigated with DSC to determine the corresponding changes in softening, melting, and crystallization temperatures. Figure 2 shows the first run DSC traces of REF and V1 to V4 granules, indicating shifts of the glass transition and melting temperatures and the magnitudes of the melting peaks. Clearly visible are the glass transition of the hard segment *T_g,hs_* around 50 °C and the broad melting peaks for PBAT around 115 °C and for PLA around 150 °C. In the cooling run, a single crystallization peak occurred around 65 °C, coinciding with the glass transition. Furthermore, a small peak at 62 °C was found in the first run due to the evaporation of tetrahydrofuran (THF), indicating chemical reactions of PBAT and PLA during compounding [40]. This peak vanished in the second run.

The evaluation of the DSC traces of the first heating, cooling, and second heating runs of REF and V1 to V4 showed 1 to 5 K lower glass transition temperatures for V1 to V4 in the first runs (Table 3). This meant that the hard segments had higher mobility due to CECL-induced reactions, which increased distances between chains or left unreacted CECL molecules acting as softeners. In the second runs, the glass transition temperatures were 7 to 11 K higher compared to the first runs, indicating further chemical reactions in the melt before starting the cooling run. With respect to REF, the *T_g,hs_* values of the second run of V1 were decreased by 1.6 K (indicating chain scission or degradation in the hard segment phase), those of V2 were unaltered (indicating no degradation), while those of V3 and V4 were increased by 3 K (indicating cross-linking).

The melting temperatures (peaks) *T_m_*_1__1 of V1 to V4 were 5 to 8 K lower than that of REF, with a *T_m_*_1__1 of 113°C, showing that CECL hinders the formation of the thicker lamellae in PBAT. In the second runs, all compounds had melting temperatures (peak) *T_m_*_1__2 of around 116 °C. The melting temperatures *T_m_*_2__1 of REF and V1 to V4 ranged between 150 and 153 °C, indicating the similar structures of the PLA phase in all compounds; however, *T_m_*_2__2 peaks were only found for REF and V2, indicating suppressed PLA crystallization in V1, V3, and V4. The fusion heats of V3 and V4 (5 to 6 J g^−1^) were smaller in both runs than for REF (8 J g^−1^), showing that cross-linking reduces crystallinity. Interestingly, the crystallization enthalpy in the cooling runs exceeded the total fusion heats (sum of both peaks) by 20 to 50%; they were expected to exceed the crystallization enthalpies. As the low temperature integration limit was chosen below *T_g,hs_*, the crystallization enthalpies increased due to a baseline effect, which included the glass transition. Determining the fusion heats using integration limits of 40 °C and 160 °C provided roughly double the Δ*H_m_*_1_.

**Figure 3:**
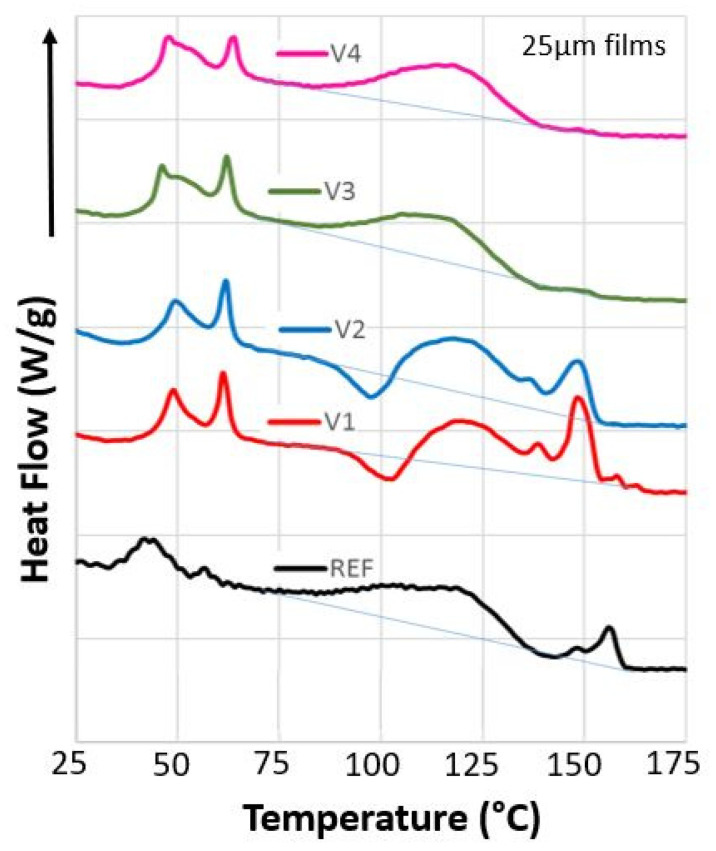
DSC traces of REF, V1 to V4, first runs (0.2 W/g) of 25 µm films with glass temperature *T_g,hs_*, the THF boiling temperature *T_THF_*, melting temperatures *T_m_*_1_ and *T_m_*_2_, and corresponding melting and crystallization peaks.

The first runs of DSC measurements of granules showed the second melting process and that chemical reactions took still place in the melt. In order to investigate how both chemical reactions and processing via film blowing change the structure of the compounds, DSC measurements of 25 µm and 100 µm films were performed as well. Their DSC traces were very similar and almost independent of the film thickness, although they differed significantly from those of the granules (Figure 3).

The glass transitions were more pronounced, indicating that the films were more amorphous due to the faster solidification during film blowing. Above *T_g,hs_*, the polymer chains in the films gain mobility, leading to shrinkage, as indicated by decreasing heat flow and the beginning of crystallization. The THF peaks of V1 to V4 were more intense, indicating that more CECL-induced reactions between PBAT and PLA occur during film blowing. V1 and V2 showed recrystallization peaks starting at 90 °C and pronounced PLA peaks. No recrystallization and PLA peaks were found for V3 and V4.

The evaluation of the DSC traces (Table 4) shows that the 100 µm films have about 9 K lower *T_g,hs_* than the 25 µm films for REF. V1 to V4 films exhibit a similar behavior as the granules; in the first heating run, V3 and V4 have lower *T_g,hs_* values than V1 and V2, whereas in the second heating run the opposite is true. This means that the amorphous hard segment phases of V3 and V4 are more disordered after film blowing, probably due to incomplete chemical reactions. Only re-melting settles the final structure, which contains highly cross-linked polymer chains.

Above *T_g,hs_* a certain exothermal heat flow is generated by free film shrinkage of the films due to relaxation of processing-induced molecular orientations [41]. These relaxations lead to a slow increase in crystallinity due to healing of structural defects. This generates recrystallization peaks for the 100 µm films of V1 and V2, which also exhibited higher MVR values and higher chain mobilities. As a consequence, the evaluated fusion heats of PBAT and PLA cannot be considered as a measure of the initial crystallinities.

The films of V1 to V4 showed pronounced THF peaks compared to the REF films, showing roughly two-fold values for 25 µm films and four-fold values for 100 µm films. This can be explained by the 16 times faster cooling of the 25 µm films, leaving significantly less time for CECL-induced reactions between PBAT and PLA, and partly by the higher draw ratio, which reduces the contact times amongst reactive groups due to chain slippage.

The 25 µm films of REF and V1 to V4 exhibited both PBAT and PLA melting peaks as well as the 100 µm films of V1 and V2, whereas no PLA peaks were found for 100 µm films of V3 and V4. If one supposes that PLA segments of highly oriented polymer chains slip along each other, they can form pre-crystalline PLA structures, promoting crystallization during film blowing. As the polymer chains of the 100 µm films of V3 and V4 were not strained sufficiently, pre-crystalline PLA structures were not formed against chain recoiling.

The crystallization enthalpies of molten films of REF and V1 to V4 were 1 to 5 J g^−1^ higher than those of the granules. This indicated that preferential chain orientations remained due to the film blowing, even in the melts, easing and enabling subsequent crystallization.

Comparing the second runs to the first runs showed how REF and V1 to V4 were affected by processing. The midpoints of *T_g,hs_* were shifted in temperature from 53 °C to 58 °C. Assuming a STD of 2 K, one can say that REF, V1, and V2 have identical *T_g,hs_* values, whereas V3 and V4 showed increased values, indicating cross-linking in the hard segment phase. The fusion heats of the first runs exceeded those of the second runs, indicating orientation relaxations after film melting. Furthermore, no or only small melting peaks of PLA occur in the second runs, supporting the crucial role of a chain orientation for the formation of the PLA crystallites. Only 25 µm films of V2 exhibited double peaks at 146 °C and 153 °C, with fusion heats of 2.3 J g^−1^ being close to 2.8 J g^−1^ for the first run. Only the 25 µm films of V2 performed well in the seal strength tests after aging; one may attribute this to the existence of a crystalline PLA phase.

**Figure 4:**
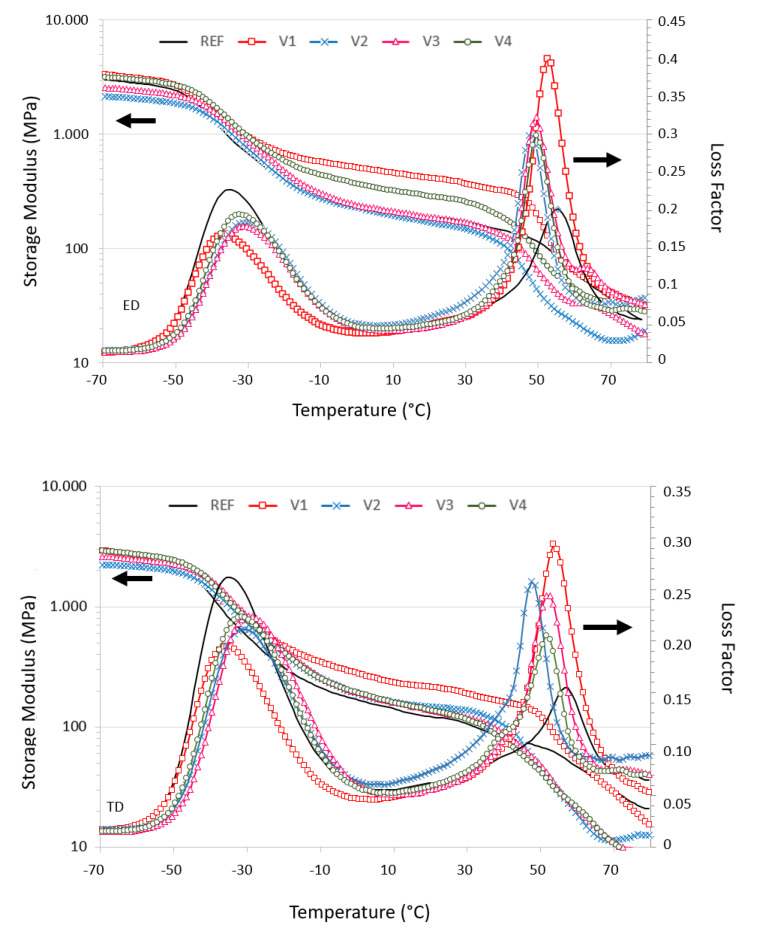
Comparison of temperature-dependent logarithmic storage moduli (log(*E*’(*T*, *ω* = 1 Hz)) and loss factor (*tanδ*) of REF and V1 to V4 in the ED and TD directions at 23 °C.

Both the introduction and processing of CECL affect the temperature-dependent storage moduli in the ED and TD in terms of glass transition temperature shifts and stiffness levels (Figure 4). The storage moduli of the 100 µm films were anisotropic (Table 5), with MD/TD ratios of REF = 1.4, V1 = 1.2, V2 = 1.4, V3 = 1.5, and V4 = 2. These were less pronounced compared to the tensile moduli of 25 µm films, with MD/TD ratios of REF = 2.5, V1 = 2.5, V2 = 3.1, V3 = 2.8, and V4 = 1.7, Table 2. The storage moduli between *T_g,ss_* and *T_g,hs_* increased in MD for V1 (factor 2) and V4 (factor 1.5), whereas they were not altered for REF, V2, or V3. In the TD, V1 exhibited an increased storage modulus (factor 2.5), whereas only slight increases were observed for V2, V3, and V4 compared to the REF; however, the moduli increases were more pronounced in the ED (maximum: factor 2.4 for V1) compared to the TD (maximum: factor 1.9 for V1) (Figure 4). This shows that CECL reactions leading to chain scission or degradation for V1 and V2 or cross-linking for V3 and V4 are strongly affected by the achieved strains of the polymer chains.

A comparison of storage moduli *E*’ to the moduli from tensile tests *E* showed that in ED, the moduli *E_ED_* (25 µm films) exceeded the storage moduli *E’* (100 µm films) by a factor 2 to 2.5, except for V1 with *E_ED_* ≈ *E’_ED_*;

In the TD, the moduli *E_TD_* (25 µm films) were between 120 and 180 MPa and were slightly larger than the storage moduli *E’* (100 µm films); only V4 resulted in *E_TD_* ≈ 3 *E’_TD_*.

This can be explained by the 4 times higher drawing ratio of the 25 µm films and shorter cooling times. DMA measurements provided the glass transition temperatures of both the soft (*T_g,ss_*) and hard (*T_g,hs_*) segment phases (Table 5). For REF and V1, the *T_g,ss_* values coincided, whereas they were shifted for V2 to V4 by 3 to 5 K towards higher temperatures, indicating cross-linking in soft segment phases. Interestingly, the *T_g,hs_* values of V1 to V4 decreased by 2 to 4 K, respectively, compared to REF. This was in contrast to the DSC data, whereby *T_g,hs_* values of V1 to V4 increased by 7 to 8 K (Table 4); however, one has to keep in mind that films shrunk freely in the DSC experiment, reducing the free volume and shifting *T_g,hs_* to higher temperatures. Shrinkage was prevented in the DMA experiment due to the sample clamps and because the free volume was maintained, at least at the beginning of the softening. As REF lacks structural disorder due to chemical reactions, its *T_g,hs_* value exceeded those of V1 and V2.

The temperature ranges in which the glass transitions of the soft segment phase took place were wider than those of the hard segment phase. The *T_g,ss_* values due to the maxima of *tanδ* were 6 to 7 K higher than the *T_g,ss_* values due to the maxima of *E’* whereas they differed by only 2 to 3 K for the *T_g,hs_* values of hard segment phases (Figure 4). This indicates that CECL reactions preferably take place in the soft segment phase.

## 4. Conclusions

The PBAT/PLA blend *M·VERA^®^ B5029* was modified with four multifunctional chain-extending cross-linkers (CECL). The introduction of CECL modified the properties of the reference PBAT/PLA blend significantly. The tris(2,4-di-tert-butylphenyl)phosphite and 1,3-phenylenebisoxazoline led to chain scission or degradation, whereas aromatic polycarbodiimide and poly(4,4-dicyclohexylmethanecarbodiimide) caused cross-linking of the modified blends. DSC data for the granules and films showed that the chemical reactions were incomplete after compounding and that film blowing intensifies them, even for 25 µm films being rapidly cooled. This behavior is explainable if CECL molecules are linked with one reactive site of polymer chains during compounding, while other reactive sites only show reaction possibilities if a chain slip occurs, such as in elongational flow during film blowing. Further, the chain scission and cross-linking are different for the extrusion direction (ED) and the direction transverse to extrusion (TD). This view explains that elongation at the break in ED and tear resistance in TD decrease with aging, whereas elongation at the break in the TD and tear resistance in the ED remain unaltered. The compounds consisted of soft and hard segment phases, and their shifts in *T_g_* with respect to the reference blend showed in which phase reactions took place. According to DMA data, tris(2,4-di-tert-butylphenyl)phosphite hardly affected the *T_g_* values of either segment, whereas other CECLs caused increases in the soft segment phase and decreases in the hard segment phase. This meant that cross-linking occurred in the soft phase, whereas CECL reactions occurred in the hard phase only with increased free volume or CECL. Finalized cross-linking in the hard segment phase was found for aromatic polycarbodiimide and poly(4,4-dicyclohexylmethanecarbodiimide) after melting of the granules and films.

In spite of the fact that different effects were seen in soft and hard segments that are caused by various CECLs, the most synergetic effect was shown by 1,3-phenylenebisoxazoline. Even though the clear degradation (increase in MVR) was detected for 1,3-phenylenebisoxazoline, this provided the most mechanically stable blend over the time compared with REF, as well as better hydrolytic behavior with temperatures close to the *T_g_* in the hard segment. 

## Figures and Tables

**Table 1:** MVR and mechanical properties of REF and modified compounds V1 to V4 in the extrusion direction (ED) and transverse direction (TD).

Code	*E_ED_*	*E_TD_*	*σ_max,ED_*	*σ_max,TD_*	*ε_break,ED_*	*ε_break,TD_*	*F_tear,ED_*	*F_tear,TD_*	*F_seal_*	MVR
	MPa	MPa	MPa	MPa	%	%	N mm^−1^	N mm^−1^	N	cm^3^ 10 min^−1^
REF	457 ± 36	181 ± 22	28 ± 3	29 ± 3	400 ± 48	450 ± 27	107 ± 3	54 ± 17	7.0 ± 0.3	4.0 ± 0.1
V1	399 ± 42	177 ± 22	31 ± 3	33 ± 4	210 ± 10	450 ± 30	123 ± 38	41 ± 13	0.4 ± 0.2	5.0 ± 0.1
V2	501 ± 31	162 ± 45	24 ± 2	24 ± 2	250 ± 31	450 ± 30	110 ± 34	35 ± 11	8.5 ± 0.3	5.8 ± 0.2
V3	407 ± 66	147 ± 5	22 ± 3	25 ± 2	250 ± 31	460 ± 16	118 ± 38	40 ± 13	8 ± 0.6	0.6 ± 0.1
V4	627 ± 56	380 ± 66	33 ± 3	35 ± 3	240 ± 14	440 ± 27	112 ± 35	40 ± 12	10 ± 2	1.0 ± 0.1

**Table 2:** MVR and mechanical properties of REF and modified compounds V1 to V4 in the extrusion direction (ED) and transverse direction (TD) aged for 1 and 2 months at 50 °C.

	Change of Properties with Respect to Corresponding Aging States									
Code	*E_ED_* (MPa)	*E_TD_* (MPa)	*σ_max,ED_* (MPa)	*σ_max,TD_* (MPa)	*ε_break,ED_* (%)	*ε_break,TD_* (%)	*F_tear,ED_* (N mm^−1^)	*F_tear,TD_* (N mm^−1^)	*F_seal_* (N)	MVR (cm^3^ 10 min^−1^)
	Aging 1 month at 50 °C									
REF	437 ± 37	238 ± 10	20.4 ± 2.6	21.8 ± 2.2	310 ± 39	390 ± 20	104 ± 33	57 ± 18	5.9 ± 0.4	4.7 ± 0.1
V1	379 ± 21	183 ± 10	20 ± 1	22 ± 2	270 ± 17	450 ± 23	139 ± 43	35 ± 11	0.4 ± 0.2	5.5 ± 0.2
V2	422 ± 20	182 ± 18	22 ± 2	23 ± 2	260 ± 17	480 ± 15	142 ± 44	31 ± 10	6.5 ± 0.3	6.3 ± 0.2
V3	453 ± 16	206 ± 11	31 ± 1	33 ± 2	200 ± 16	450 ± 32	132 ± 41	30 ± 10	0.6 ± 0.1	0.4 ± 0.1
V4	454 ± 38	265 ± 33	30 ± 1	31 ± 1	220 ± 11	420 ± 19	111 ± 35	29 ± 9	0.9 ± 0.1	1.4 ± 0.1
	Aging 2 months at 50 °C									
REF	363 ± 48	197 ± 12	21.2 ± 1.5	26.1 ± 1.8	410 ± 26	500 ± 20	80 ± 25	51 ± 16	4.8 ± 0.6	5.5 ± 0.2
V1	400 ± 11	187 ± 23	20 ± 2	21 ± 1	250 ± 24	430 ± 14	138 ± 43	31 ± 13	0.6 ± 0.3	5.9 ± 0.2
V2	436 ± 26	207 ± 20	32 ± 2	36 ± 2	200 ± 20	480 ± 39	130 ± 30	32 ± 11	5.5 ± 0.9	7.9 ± 0.3
V3	436 ± 37	207 ± 14	32 ± 3	36 ± 3	200 ± 32	480 ± 29	112 ± 35	30 ± 10	0.5 ± 0.1	0.35 ± 0.1
V4	460 ± 27	236 ± 25	30 ± 2	29 ± 3	210 ± 10	430 ± 33	109 ± 34	43 ± 14	-	1.2 ± 0.1

**Table 3:** The glass temperatures of hard segments *T_g,hs_*, crystallization temperature *T_cr_*, and melting temperatures *T_m_*_1_ of PBAT and *T_m_*_2_ of PLA with corresponding fusion heat values Δ*H_m_*_1_ and Δ*H_m_*_2_, as determined in the first run, cooling run, and second run of the DSC measurements of granules of REF and V1 to V4 compounds.

Properties	Unit		REF	V1	V2	V3	V4
*T_g,hs_*_1	°C	Onset	50.0	43.7	46.7	47.8	46.2
		Midpoint	54.1	49.0	49.8	52.8	49.7
*T_m_*_1__1	°C	Onset	81.2	84.8	77.8	94.9	81.1
		Peak	113.4	105.8	108.7	107.6	105.1
*T_m_*_2__1	°C	Onset	143.2	144.2	143.4	142.7	143.2
		Peak	149.9	152.0	152.7	149.9	152.7
*T_cr_*	°C	Onset	75.4	83.4	83.0	87.3	87.2
		Peak	65.3	72.6	71.9	76.2	75.2
*T_g,hs_*_2	°C	Onset	55.9	51.1	54.9	58.4	59.2
		Midpoint	57.5	55.9	57.2	60.8	60.8
*T_m_*_1__2	°C	Onset	88.4	89.1	88.9	83.6	83.8
		Peak	115.6	116.6	115.2	116.3	116.6
*T_m_*_2__2	°C	Onset	143.8	-	144.0	-	-
		Peak	146.6	-	149.2	-	-
Δ*H_m_*_1__1	J g^−1^		8.5	9.4	6.9	5.1	5.2
Δ*H_m_*_2__1	J g^−1^		0.3	1.1	1.9	1.1	2.8
Δ*H_cr_*			11.0	11.6	10.9	9.1	10.3
Δ*H_m_*_1__2	J g^−1^		7.3	7.3	5.8	5.8	5.8
Δ*H_m_*_2__2	J g^−1^		0	0	0.8	0	0

Note: Numbers following an underline indicate the first or second run. The temperature accuracy is ±1 K.

**Table 4:** The glass temperatures of hard segments *T_g,hs_*, THF boiling temperature *T_THF_*, THF boiling enthalpy Δ*H_THF_*, and fusion heats Δ*H_m_*_1_ (PLA) and Δ*H_m_*_2_ (PBAT) as determined by the first run and crystallization enthalpy Δ*H_cr_* of 25 µm and 100 µm films of REF and V1 to V4 compounds.

Code	*T_g,hs_* (°C)			*T_THF_* (J g^−1^)	Δ*H_THF_* (J g^−1^)	Δ*H_m_*_1_ (J g^−1^)	Δ*H_m_*_2_ (J g^−1^)	Δ*H_cr_* (J g^−1^)
	Onset	Midpoint	Endset			PBAT	PLA	
25 µm film								
REF	43.0	47.4	52.2	60.5	0.2	5.4	1.1	15.2
STD	0.7	0.0	0.5	0.4	0.1	0.4	0.1	0.9
V1	44.9	47.6	49.3	61.3	0.3	5.4	1.2	15.1
STD	0.6	0.0	1.8	0.8	0.1	0.5	0.1	0.1
V2	44.4	48.5	51.8	63.9	0.5	5.1	2.9	14.7
STD	1.7	0.4	0.6	0.1	0.0	0.2	0.1	0.1
V3	41.7	45.3	48.4	63.8	0.4	6.4	1.2	11.7
STD	0.4	0.3	0.1	0.7	0.0	0.1	0.0	0.3
V4	43.3	46.4	50.4	63.1	0.3	3.7	3.5	12.7
STD	0.9	0.7	0.0	0.1	0.1	0.0	0.1	0.6
100 µm film								
REF	34.2	38.3	42.2	56.4	0.2	9.5	1.4	15.2
STD	1.1	0.4	0.1	0.3	0.1	1.8	0.1	0.3
V1	41.4	46.2	48.9	61.4	1.0	5.8	2.6	13.5
STD	0.4	0.1	0.3	0.1	0.0	0.2	0.1	0.6
V2	43.9	46.0	48.4	61.7	0.9	4.2	1.7	10.4
STD	1.3	0.8	0.4	0.1	0.0	0.4	0.1	0.1
V3	43.5	44.8	45.7	62.2	0.7	7.2	0.0	15.6
STD	0.1	0.1	0.1	0.1	0.0	0.2	0.0	0.1
V4	44.0	45.5	47.0	63.0	0.7	8.0	0.0	14.7
STD	0.6	0.8	0.5	0.8	0.0	0.4	0.0	0.4

**Table 5:** The storage moduli *E’* and loss moduli *E”*, glass temperatures of soft segments *T_g,ss_*, and glass temperatures of hard segments *T_g,hs_* determined using the maxima of *E”* and *tan δ* for REF and V1 to V4 compounds measured at 23 °C with a frequency of 1 Hz.

Sample		*E’* (MPa)	*E”* (MPa)	*T_g,ss_* (*E*”) (°C)	*T_g,hs_* (*E*”) (°C)	*T_g,ss_* (*tanδ* °C)	*T_g,hs_* (*tanδ* °C)
REF	ED	177 ± 33	10.3 ± 1.2	−41.7 ± 0.2	52.4 ± 0.8	−35.5 ± 0.1	54.8 ± 0.7
	TD	124 ± 13	8.0 ± 1	−41.9 ± 0.1	55.0 ± 0.4	−35.2 ± 0.1	56.7 ± 0.3
V1	ED	387 ± 38	21.0 ± 1.4	−41.7 ± 0.6	50.6 ± 1.2	−36.8 ± 0.9	53.3 ± 1.2
	TD	322 ± 17	20.0 ± 1.6	−41.9 ± 0.1	51.0 ± 0.1	−36.1 ± 0.3	53.6 ± 0.1
V2	ED	210 ± 33	13.0 ± 1.4	−38.8 ± 0.7	50.0 ± 4.2	−32.7 ± 0.5	51.9 ± 4.5
	TD	151 ± 12	11.0 ± 0.8	−38.9 ± 0.9	50.0 ± 1.3	−32.2 ± 0.9	52.1 ± 1.1
V3	ED	184 ± 15	9.3 ± 1.2	−36.8 ± 0.7	50.5 ± 5.8	−31.0 ± 0.5	52.5 ± 6.2
	TD	126 ± 5	7.3 ± 0.5	−37.2 ± 0.5	53.6 ± 3.8	−30.6 ± 0.5	56.1 ± 4.0
V4	ED	270 ± 24	15.0 ± 1.9	−38.7 ± 1.3	50.0 ± 4.3	−32.9 ± 1.9	52.0 ± 4.4
	TD	133 ± 6	8.7 ± 0.9	−38.0 ± 0.4	49.6 ± 5.7	−31.8 ± 0.8	51.8 ± 6.1

## Data Availability

Data are stored at the personal depository of J. Azevedo.
